# Genotype effects contribute to variation in longitudinal methylome patterns in older people

**DOI:** 10.1186/s13073-018-0585-7

**Published:** 2018-10-22

**Authors:** Qian Zhang, Riccardo E Marioni, Matthew R Robinson, Jon Higham, Duncan Sproul, Naomi R Wray, Ian J Deary, Allan F McRae, Peter M Visscher

**Affiliations:** 1 Institute for Molecular Bioscience, The University of Queensland, Brisbane, QLD 4072 Australia; 2 Centre for Cognitive Ageing and Cognitive Epidemiology, University of Edinburgh, Edinburgh, EH8 9JZ UK; 3 Medical Genetics Section, Centre for Genomic and Experimental Medicine, Institute of Genetics & Molecular Medicine, University of Edinburgh, Edinburgh, EH4 2XU UK; 4 Medical Research Council Human Genetics Unit, Medical Research Council Institute of Genetics and Molecular Medicine, University of Edinburgh, Edinburgh, EH4 2XU UK; 5 Edinburgh Cancer Research Centre, Medical Research Council Institute of Genetics and Molecular Medicine, University of Edinburgh, Edinburgh, EH4 2XU UK; 6 The Queensland Brain Institute, The University of Queensland, St Lucia, QLD 4072 Australia; 7 Department of Psychology, University of Edinburgh, Edinburgh, EH8 9JZ UK

**Keywords:** DNA methylation, Longitudinal analysis, Methylation change, G by AGE

## Abstract

**Background:**

DNA methylation levels change along with age, but few studies have examined the variation in the rate of such changes between individuals.

**Methods:**

We performed a longitudinal analysis to quantify the variation in the rate of change of DNA methylation between individuals using whole blood DNA methylation array profiles collected at 2–4 time points (*N* = 2894) in 954 individuals (67–90 years).

**Results:**

After stringent quality control, we identified 1507 DNA methylation CpG sites (rsCpGs) with statistically significant variation in the rate of change (random slope) of DNA methylation among individuals in a mixed linear model analysis. Genes in the vicinity of these rsCpGs were found to be enriched in Homeobox transcription factors and the Wnt signalling pathway, both of which are related to ageing processes. Furthermore, we investigated the SNP effect on the random slope. We found that 4 out of 1507 rsCpGs had one significant (*P* < 5 × 10^−8^/1507) SNP effect and 343 rsCpGs had at least one SNP effect (436 SNP-probe pairs) reaching genome-wide significance (*P* < 5 × 10^−8^). Ninety-five percent of the significant (*P* < 5 × 10^−8^) SNPs are on different chromosomes from their corresponding probes.

**Conclusions:**

We identified CpG sites that have variability in the rate of change of DNA methylation between individuals, and our results suggest a genetic basis of this variation. Genes around these CpG sites have been reported to be involved in the ageing process.

**Electronic supplementary material:**

The online version of this article (10.1186/s13073-018-0585-7) contains supplementary material, which is available to authorized users.

## Background

DNA methylation is a widely studied epigenetic modification with a role in the regulation of gene expression [1]. Local levels of DNA methylation differ within and between individuals. This variation in local methylation is associated with both genetic and environmental factors [2–5]. The majority of DNA methylation studies in human are based on cross-sectional cohorts. Such studies have reported that methylation levels at many CpG sites in the genome correlate with age [6–10]. Therefore, age is frequently treated as a covariate and adjusted for in a linear regression framework in which differences of DNA methylation between cell types, tissues and diseases are tested [11–13]. One implicit assumption behind this correction is that the rate of change at a methylation CpG site across time is constant between individuals, which may not be true. Several studies have revealed that there is a potential change in variability of DNA methylation with age [14, 15], indicating that the rate of change of DNA methylation is different between individuals.

Estimation of the variation in such trajectories of DNA methylation with age between individuals is possible in a longitudinal analysis. Previous longitudinal analyses have investigated the relationship between SNPs and longitudinal DNA methylation [16, 17]. Other studies focused on the association between DNA methylation and a phenotype measured on the same individual [18–20]. Differences between individuals in the pattern of change of DNA methylation over time were not considered in these studies. Here, we estimate the variation in trajectories of DNA methylation change among individuals in a longitudinal analysis. This approach may elucidate how epigenetic marks change differently between individuals and whether this variation is associated with genetic factors and biological function.

In this study, we estimated between-individual variation in the rate of change of DNA methylation at 344,000 loci in a longitudinal sample of older people from the Lothian Birth Cohorts 1921 and 1936. For each CpG site, we estimated the variation in the rate of change in each individual. Furthermore, the identification of such probes facilitates the estimation and partitioning of the variation underlying DNA methylation changes, for example, the contribution of genetic factors. We identified genetic loci that are associated with differences in the longitudinal changes in DNA methylation across individuals.

## Methods

### Methylation data

DNA was extracted from whole blood samples in Lothian Birth Cohort 1921 (LBC1921) at MRC Technology, Western General Hospital, Edinburgh (LBC1921), and the Wellcome Trust Clinical Research Facility (WTCRF), Western General Hospital, Edinburgh (LBC1936), using standard methods. Methylation typing of 485,512 probes was performed at the WTCRF. Bisulphite converted HD Methylation protocol and Tecan robotics (Illumina). Raw intensity data were background-corrected and normalized using internal controls, and methylation *M* values were generated using the R minfi package [21]. Detailed further quality control steps are given in Additional file 1.

### Batch and covariate adjustment

Our analysis was based on the *M* value of DNA methylation. We regularized the *M* value by constraining it to be in the interval between − 9.96 and 9.96 (corresponding to the interval 0.001 to 0.999 of the beta-value). Furthermore, for each probe, we removed individuals with DNA methylation three standard deviations above and below the mean *M* value to exclude outliers. On average, 34 (out of 2894 samples, 1.2%) outliers were removed for each probe. DNA methylation (*M* value) in most (79.5%) of these outliers is in the range between − 6.6 and 6.6 (corresponding to the interval 0.01 to 0.99 of the beta-value), suggesting the “abnormal” DNA methylation values in the majority of outliers are not extreme values caused by the transformation from beta-value to *M* value. Covariates including sex, age and cell counts (CC), and batch effects including position in array (PIA), hybridization date (HD), set ID (SI), plate ID (PI) and array ID (AI, both PI and AI were regarded as random effects), were corrected for each probe. We used the residuals after this adjustment for further analysis. If *y*_*j*_ is the DNA methylation value for probe *j*, then we used the residuals from the model

$$ {y}_j\sim \mathrm{sex}+\mathrm{age}+\mathrm{CC}+\mathrm{PI}\mathrm{A}+\mathrm{HD}+\mathrm{SI}+\mathrm{PI}+\mathrm{AI}+{e}_j. $$

### LBC genotype and imputation

Individuals from LBC1921 and LBC1936 were genotyped on Illumina 610-Quad Beadchip arrays. Full details of genotyping procedures are given elsewhere [22]. Standard QC filters were applied, and remaining genotyped SNPs were phased using HAPI-UR [23] and imputed using 1000 Genomes Phase I Version 3 [24] with Impute V2 [25]. Raw imputed SNPs were filtered to remove any SNPs with low imputation quality as defined by an *R*^2^ < 0.8. Subsequent quality control removed SNPs with MAF < 0.01, those with HWE *P* < 1 × 10^−6^ and a missing rate > 10%. After filtering, 7,760,689 SNPs remained for further analysis.

### Estimation of random slope effects

For each probe, we fitted a mean level and a rate of change of DNA methylation for each individual and tested whether the variance due to these random effects was significantly larger than zero, using the mixed model

$$ {y}_{ij}={u}_1+{u}_2\times {t}_{ij}+{a}_i+{b}_i\times {t}_{ij}+{e}_{ij} $$

$$ \left[\begin{array}{c}{a}_i\\ {}{b}_i\end{array}\right]\sim N\left(0,\Omega \right)\;\mathrm{with}\;\Omega =\left[\begin{array}{cc}{\sigma}_a^2& {\sigma}_{ab}\\ {}{\sigma}_{ab}& {\sigma}_b^2\end{array}\right]\;\mathrm{and}\;{e}_{ij}\sim N\left(0,{\sigma}_e^2\right). $$

where *y*_*ij*_ is the methylation residual after QC steps, *i* is the *i*th individual, *j* represents the *j*th observation in individual *i*, *u*_1_ is the mean effect, *u*_2_ is the mean age effect, *a*_*i*_ and *b*_*i*_ are the random intercept (mean level of DNA methylation in each individual) and random slope, *t*_*ij*_ represents standardized age (mean = 0 and variance = 1) and *e*_*ij*_ is the random error. The random effects of e_*ij*_, *a*_*i*_ and *b*_*i*_ are assumed to follow a normal distribution. ${\sigma}_a^2$ and ${\sigma}_b^2$ are the variances of *a*_*i*_ and *b*_*i*_, respectively. *σ*_*ab*_ is the covariance between *a*_*i*_ and *b*_*i*_, it was set to be zero under the assumption of independence between *a*_*i*_ and *b*_*i*_. The likelihood ratio test (LRT) was used to test if ${\sigma}_a^2$ and ${\sigma}_b^2$ are equal to zero.

We obtained a *P* value from the LRT using a *χ*^2^(1) distribution and then dividing the *P* value by two. This can be justified since under the null hypothesis, in 50% of cases, the test statistic is zero (or, follows a *χ*^2^(0)), and in 50% of cases it follows a *χ*^2^(1) [26]. Probes with a *P* value smaller than 1.5 × 10^−7^ (0.05/344,000) for the random slope are defined as rsCpG.

### Covariance between *a*_*i*_ and *b*_*i*_

We applied an extended model that included the covariance *σ*_*ab*_ between the random intercept and random slope to quantify the effect of covariance on the estimation of random effects. We calculated the Pearson corrections between the estimated random slope effects before and after incorporating the covariance term. All correlations were larger than 0.7 in the 1507 rsCpGs (mean correlation = 0.93). These results indicated that the introduction of the covariance term did not alter the results substantially.

### Quadratic effect

We investigated the effect of modelling a quadratic average trajectory by adding a squared term for age (*t*^*2*^) in the model ${y}_{ij}={u}_1+{u}_2\times {t}_{ij}+{a}_i+{b}_i\times {t}_{ij}+{t}_{ij}^2+{e}_{ij}$. All the correlations of the probes with and without fitting this additional term were found to be larger than 0.98 in 1507 rsCpGs.

### Estimating the confidence interval of the correlation based on bootstrapping

Considering the background correlation of DNA methylation between CpG sites, we used bootstrapping to calculate the 95% confidence interval of the correlation (of the estimated variances) between two groups of individuals. We resampled 344,000 pairs of variances with replacement from the original data and estimated the correlation based on these pairs. We repeated this step 30,000 times to calculate the 95% confidence interval of the correlation.

### GWAS on random effects

Based on the random effects estimated from the above mixed linear model, we performed a series of genome-wide association studies by using the random effects as the dependent variables with the software PLINK2 [27]. All QC-ed SNPs were used, and *P* value threshold for the significance was Bonferroni corrected (*P* < 5 × 10^−8^/1507).

### Permutation analysis of the random slope test statistics

The mean and median test statistic across CpG sites for the effect of the random slope was very large, with a *λ* inflation value (mean test statistic) of 11.0. To verify if the results are inflated under the null hypothesis, we permuted ages across individuals and waves 500 times. For each round, we re-fitted the full model on the permuted data, and the mean chi-square among the probes was calculated. The mean of this distribution was around 0.73 (SD = 0.32), which shows no significant difference (*P* = 0.48) with the expected value of 0.5 under the null hypothesis. This indicates the statistical significance of the estimated effects of a random slope is not caused by the violations of the assumptions of the distribution of the test statistic under the null hypothesis.

### Mapping CpG Islands and differently methylated region (DMR)

Genomic positions of the CpG island were obtained from the UCSC Genome Browser [28]. Annotation information of the differently methylated region (DMR) was from the Illumina DNA methylation annotation file (GEO ID GPL13534). The significance of enrichment analysis was assessed by permutation.

### PANTHER over-representation test

We used all 25,537 genes that are adjacent to the QC-ed 344,000 probes as the background. Eighteen thousand six hundred seven of the genes overlapped with the gene list in the PANTHER database. Ten thousand twenty out of 1235 rsCpG nearby genes were in the PANTHER database. The enrichment test was based on Fisher’s exact test, and protein classes with a false discovery rate (FDR) smaller than 0.05 were selected.

### Heritability of probes

We utilized the heritability of the significant probes estimated in the Brisbane Systems Genetics Study (BSGS) cohort [29, 30] to validate the genetic contribution to these probes. The significance of the difference from the null distribution of the mean heritability was estimated based on the average heritability of 1507 randomly selected probes from 30,000 permutation tests.

### SNP by age effect on DNA methylation

The SNP effect on random slope can be defined as slope_i_ = *β*_*k*_ × *d*_*ik*_ + *e*_*i*_, *i* is the *i*th individual, *β*_*k*_ is the effect size of SNP *k* on random slope, and *d*_*ik*_ is the dosage of SNP *k* in individual *i*. Since the DNA methylation of individual *i* at time point *t*_*ij*_ is *y*_*ij*_ = slope_*i*_ × *t*_*ij*_ + *e*_*ij*_ = (*β*_*k*_ × *d*_*ik*_ + *e*_*i*_) × *t*_*ij*_ + *e*_*ij*_ = *β*_*k*_ × *d*_*ik*_ × *t*_*ij*_ + *e*_*i*_ × *t*_*ij*_ + *e*_*ij*_ (main effects are ignored here), the SNP effect on random slope can be interpreted as SNP by age effect on DNA methylation. To compare the power in detecting these two effects, we simulated 3000 individuals, each with three age points sampled at round 60, 70 and 80 years old. DNA methylation of individual *i* at time point *t*_*ij*_ was simulated by using *y*_*ij*_ = (*β*_*k*_ × *d*_*ik*_ + *e*_*i*_) × *t*_*ij*_ + *e*_*ij*_. *d*_*ik*_ was sampled from (0,1,2) assuming Hardy–Weinberg equilibrium, the minor allele frequency of the SNP ranges from 0.05 to 0.5, the random error *e*_*ij*_ and random slope *e*_*i*_ (not explained by SNP) are assumed to follow a standard normal distribution. The effect size *β*_*k*_ of the SNP on the random slope was simulated in two ways: (1) *β*_*k*_ was sampled from a uniform distribution in the range of − 0.1 to 0.1 and (2) *β*_*k*_ was set to zero. For each type of data, we obtained *P* values in three ways: (1) from association between the SNP and the estimated random slope from a mixed linear model, (2) from association between DNA methylation and a SNP by age effect and (3) from association between DNA methylation and a SNP by age effect with random slope fitted as a covariate. We repeated this simulation 300,000 times and compared *P* values and median chi-square (*λ*_median_) between these associations. Based on the simulated data with no SNP effect on the random slope (*β*_*k*_ = 0), we found the *P* values from the second analysis method were inflated (*λ*_median_ = 2.01). *P* values from the other two ways were not inflated (*λ*_median_ = 1.00), and no difference in the detecting power was identified between these two ways based on the simulated data with SNP effect on the random slope (*β*_*k*_~*U*(− 0.1,0.1)).

### Linkage disequilibrium (LD) clumping

We applied LD clumping on the GWAS significant SNPs using PLINK2 [27], and imputed LBC genotype data was used as the reference. For each significant (*P* < 5 × 10^−8^) SNP, LD (*R*^2^) between this SNP and other significant (*P* < 5 × 10^−8^) SNPs within 1 Mbp distance were calculated and SNPs with LD larger than 0.1 were defined as a clump. Within each clump, only the SNP with smallest *P* value would be selected during LD clumping.

All analysis was performed by using R package, version 3.2.2 [31]. Figures were generated using ggplot2 [32].

## Results

### Data

DNA methylation was measured on individuals from Lothian Birth Cohort 1921 (LBC1921) and Lothian Birth Cohort 1936 (LBC1936) [33, 34] using Illumina Infinium HumanMethylation450K BeadChip arrays. There were 3471 samples across all waves of data collection (Additional file 1: Table S1), and 344,000 DNA methylation probes remained after removing probes encompassing SNPs annotated by Illumina (GEO ID GPL13534) and probes identified as potentially cross-hybridizing [35] (see details of quality control steps in Additional file 1). Only individuals with DNA methylation measured at two or more different time points were considered, and samples with inconsistent measurements (match rate < 0.8) of control probes within individuals were removed (Additional file 2: Figure S1), leaving 2894 samples from 954 individuals (Table 1). Among them, 283 individuals had DNA methylation measured at two time points, and 356 and 315 individuals were measured at three and four time points, respectively. The effects of covariates (sex, age and cell counts) and batches (position in the array, hybridization date, set ID, plate ID and array ID) on DNA methylation were removed before further analysis (‘Methods’, Additional file 1: Figures S2 and S3).

**Table 1:** Description of the DNA methylation samples in the LBC cohorts, for individuals with DNA methylation measured in at least two waves

Cohort wave	Mean age (SD)	Age range	Female	Male	Total
LBC1921W1	79.1 (0.6)	(77.9,80.6)	77	63	140
LBC1921W3	86.6 (0.4)	(85.8,87.5)	82	71	153
LBC1921W4	90.2 (0.1)	(90,90.6)	42	36	78
LBC1936W1	69.6 (0.8)	(67.7,71.3)	326	359	685
LBC1936W2	72.5 (0.7)	(70.9,74.2)	353	399	752
LBC1936W3	76.3 (0.7)	(74.7,77.7)	284	312	596
LBC1936W4	79.3 (0.6)	(78.0,80.9)	240	250	490

### Identification of CpG sites with a random slope in methylation

**Fig. 1:**
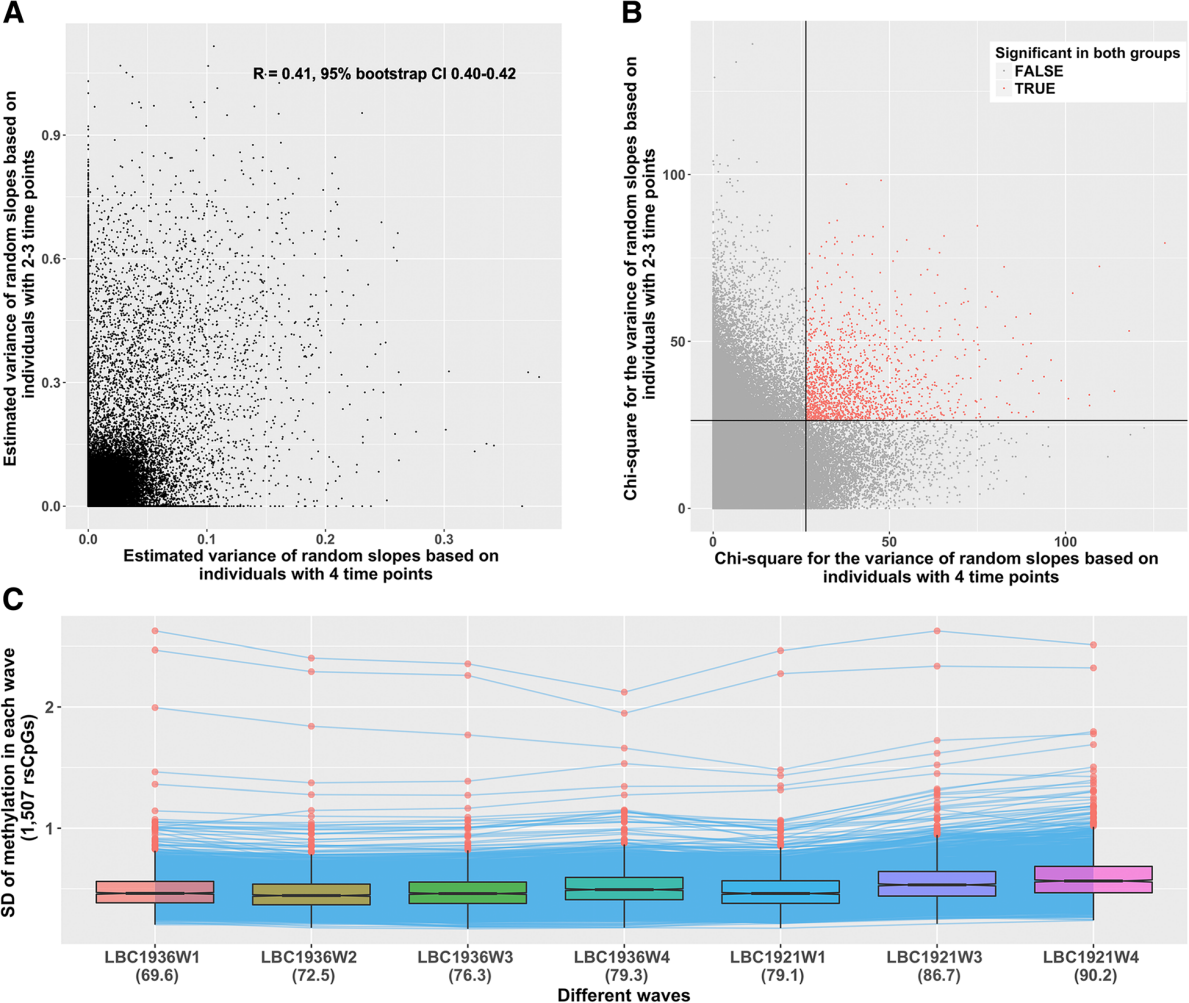
**a** Comparison of estimated variances of random slopes between the group of individuals with four time points and the group of individuals with two or three time points. **b** Comparison of chi-square test statistics for the variance of random slope between the group of individuals with four time points and the group of individuals with two or three time points. **c** The change of standard deviation (SD) in 1507 rsCpG across waves (mean age in each wave in parentheses). Each point represents the SD of DNA methylation for one CpG site in each wave, and the SD of each CpG site in different waves are connected by lines. The overall level of SD across all CpG sites in each wave is shown as a boxplot. The red dashed line is the median SD in wave 1 of LBC1936

For each CpG site, we estimated the variance of the rate of change (random slope) between individuals in a mixed linear model (‘Methods’). A non-zero variance indicates the existence of individual differences in the rate of change in DNA methylation across time. Forty-two thousand two hundred fifty-three probes were found to have a statistically significant random slope (likelihood ratio test, *P* < 0.05/344,000, Bonferroni corrected) based on 2894 samples. Permutation test analyses indicated that the statistical significance of the estimated effects of the random slope is not caused by the violations of the assumptions of the test statistic (‘Methods’, Additional file 2: Figures S4 and S5). Moreover, no substantial impact on the estimation of random effects was found by introducing a covariance (‘Methods’, Additional file 2: Figure S6A) between the random slope and the mean level of DNA methylation in each individual into the model, or the inclusion of additional corrections for age effects such as a quadratic term (‘Methods’, Additional file 2: Figure S6B). To obtain a robust set of CpG sites with a statistically significant variation in the rate of change, we divided the individuals into two groups according to the number of time points for which they have a measurement. One group contains individuals with two or three time points, and the other group has individuals with four time points. We applied the mixed linear model on each of these two groups and found that the estimated variances of random slopes for each CpG site were correlated between these independent groups (*R* = 0.41, 95% bootstrap CI 0.40–0.42, bootstrapping was repeated 30,000 times, ‘Methods’, Fig. 1a). One thousand five hundred seven CpG sites were identified to have a statistically significant (*p* < 0.05/344,000) variation in the rate of change of DNA methylation in both groups (rsCpGs, Fig. 1b, Additional file 3: Table S2). The overlap is statistically significant (odds ratio = 4.9, *P* < 3.3 × 10^−5^, permutation test, 30,000 times), and these 1507 rsCpGs were used for further analysis. A summary of chi-square statistics for the variance of the random slope is presented in Table 2.

**Table 2:** The summary of chi-square statistics for the variance of random slope in different groups of individuals

	*λ*_mean_/*λ*_median_	Number of probes with significant random slopes	Largest *χ*^*2*^	Proportion of zero *χ*^*2*^ (*χ*^*2*^ < 10^−5^) (%)	*λ*_mean_/*λ*_median_ of non-zero *χ*^*2*^
All individuals	11.0/13.9	42,253	206.2	21.3	14.0/22.1
Individuals with 2 or 3 time points	7.9/9.6	20,291	139.1	21.3	10.0/15.5
Individuals with 4 time points	3.8/1.9	6729	128.2	30.6	5.4/5.9

We observed a larger variation in DNA methylation of rsCpGs compared to randomly selected probes in each wave (Additional file 2: Figure S7). Moreover, an increase in the variability of DNA methylation with age can be identified in most of these rsCpGs (Fig. 1c). These rsCpGs overlapped with probes identified by Slieker and colleagues [14]. Based on a cross-sectional study, Slieker et al. identified 6366 positions that showed changes in variably of methylation with age using 3295 whole blood DNA methylation profiles. Among those positions, 540 probes overlap with the 1507 rsCpGs in our study. This highly significant overlap (odds ratio = 45.9, *P* < 3.3 × 10^−5^, permutation test, 30,000 times) provides an independent confirmation of our results.

### Genomic locations of CpG sites with random effects

**Fig. 2:**
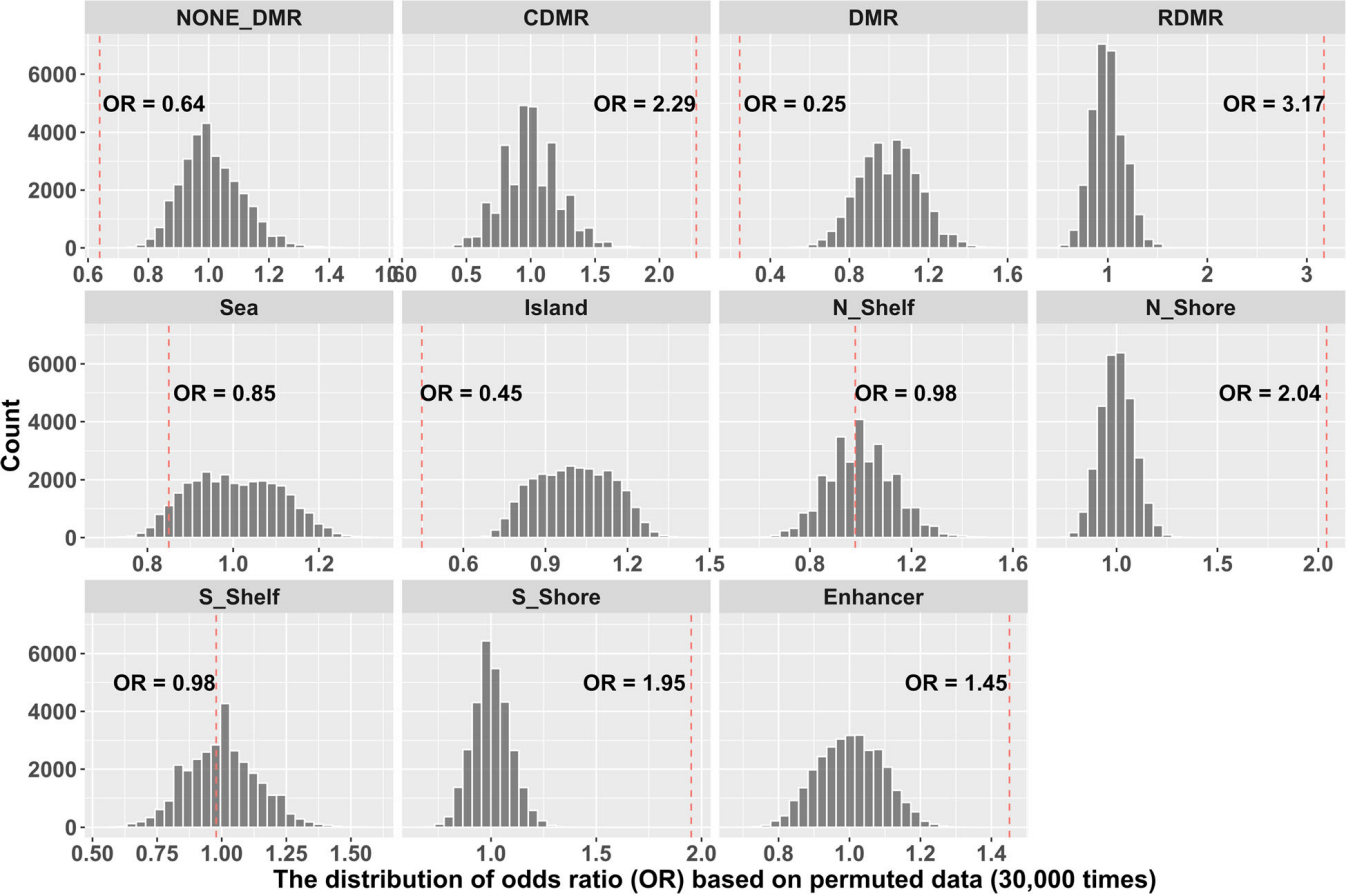
Enrichment analysis of rsCpGs in different CpG regions based on the permutation test. For each CpG region, the distribution of odds ratio based on permuted data (30,000 times) and the odds ratio based on the original data (red dashed line) are presented. DMR: differentially methylated region; CDMR: cancer-specific DMR; RDMR: reprogramming-specific DMR; NONE_DMR other CpGs not in DMR. Island: CpG island provided by UCSC [28]; N_Shore: 0–2 Kb upstream of CpG island; S_Shore: 0–2 Kb downstream of CpG island; N_Shelf: 2–4 Kb upstream of CpG island; S_Shelf: 2–4 Kb downstream of CpG island; Sea: 4 Kb away from CpG island. Enhancer: Predicted enhancer elements determined by ENCODE Consortium [46]

The dynamicity of DNA methylation varies across the human genome [36, 37]. To investigate whether the rsCpGs locate in the more variable genomic regions, we mapped these CpG sites to the genome and applied an enrichment test on these probes (‘Methods’). We observed an enrichment of rsCpGs in the Shore region of CpG islands (regions within 2 kb upstream or downstream of a CpG island are called north shore and south shore, respectively). Genomic positions of the CpG island were obtained from the UCSC Genome Browser [28]), with an odds ratio (OR) of 2.0 for both the north shore (*p* < 3.3 × 10^−5^, permutation test, 30,000 times) and the south shore (*P* < 3.3 × 10^−5^, permutation test, 30,000 times) (Fig. 2). DNA methylation in the shore region was previously reported to be more dynamic than that in CpG islands [36], and our results indicate that CpG sites with random slopes locate in the regions with more malleable DNA methylation. Similarly, rsCpGs were found to be enriched in reprogramming-specific differently methylated regions (RDMR, regions differentially methylated in the reprogramming process) [37].

### Biological enrichment of CpG sites with random effects

To explore the biological function of the rsCpGs, we applied a gene over-representation test on the nearest genes of rsCpGs using PANTHER (version 13.1) [38] (‘Methods’). The result showed that 1235 genes around the 1507 rsCpGs were statistically significantly (Fisher’s exact test, *P* = 3.7 × 10^−10^, FDR = 3.9 × 10^−8^) enriched in Homeodomain (Homeobox) transcription factor (PC00119) protein class (Table 3). We also investigated the significance of these protein classes using a permutation test and found they remained significant (100 repeats). Furthermore, we performed Gene Ontology (GO) analysis on these genes and found that ectoderm development (GO 0007398, *P*= 5.9 × 10^−18^, FDR = 1.5 × 10^−15^) and developmental process (GO 0032502, *P* = 1.8 × 10^−17^, FDR = 2.2 × 10^−15^) were the most significantly over-represented biological processes (Additional file 4: Table S3). Cadherin signalling pathway (P00012), Wnt signalling pathway (P00057) and Heterotrimeric G-protein signalling pathway-Gq alpha and Go alpha-mediated pathway (P00027) were found to be significantly enriched pathways for genes around rsCpGs in a PANTHER pathway analysis (Table 3). Since there are only 177 primarily signalling pathways in PANTHER database [38], we further performed the pathway analysis in an integrated pathway database ConsensusPathDB (version 33) [39]. This analysis on the same gene sets showed that the most significant pathway for rsCpGs was “Neuronal System” (*P* = 1.4 × 10^−9^). Full details of significant pathway results are given in the Additional file 5: Table S4.

**Table 3:** Gene enrichment test on the 1235 genes around the 1507 rsCpGs. Only protein classes with FDR smaller than 0.05 are listed

	Reference genes (18607)	Test genes	Expected genes	Over/under	Fold enrichment	Raw *P* value	FDR
PANTHER protein class							
Homeodomain transcription factor (PC00119)	101	27	5.5	+	4.9	3.7 × 10^−11^	3.9 × 10^−8^
Basic helix-loop-helix transcription factor (PC00055)	76	13	4.2	+	3.1	6.7 × 10^−4^	2.4 × 10^−2^
Helix-turn-helix transcription factor (PC00116)	176	36	9.7	+	3.7	3.1 × 10^−10^	6.6 × 10^−8^
G-protein coupled receptor (PC00021)	250	31	13.7	+	2.3	1.0 × 10^−4^	4.5 × 10^−3^
Receptor (PC00197)	644	71	35.3	+	2.0	1.7 × 10^−7^	1.2 × 10^−5^
Transcription factor (PC00218)	1073	95	58.8	+	1.6	1.2 × 10^−5^	6.6 × 10^−4^
PANTHER pathway							
Cadherin signalling pathway (P00012)	157	27	8.6	+	3.1	1.0 × 10^−6^	1.7 × 10^−4^
Wnt signalling pathway (P00057)	307	39	16.8	+	2.3	6.3 × 10^−6^	5.2 × 10^−4^
Heterotrimeric G-protein signalling pathway-Gq alpha and Go alpha-mediated pathway (P00027)	123	18	6.7	+	2.7	3.8 × 10^−4^	1.6 × 10^−2^

### Genetic effects on the random slope

**Fig. 3:**
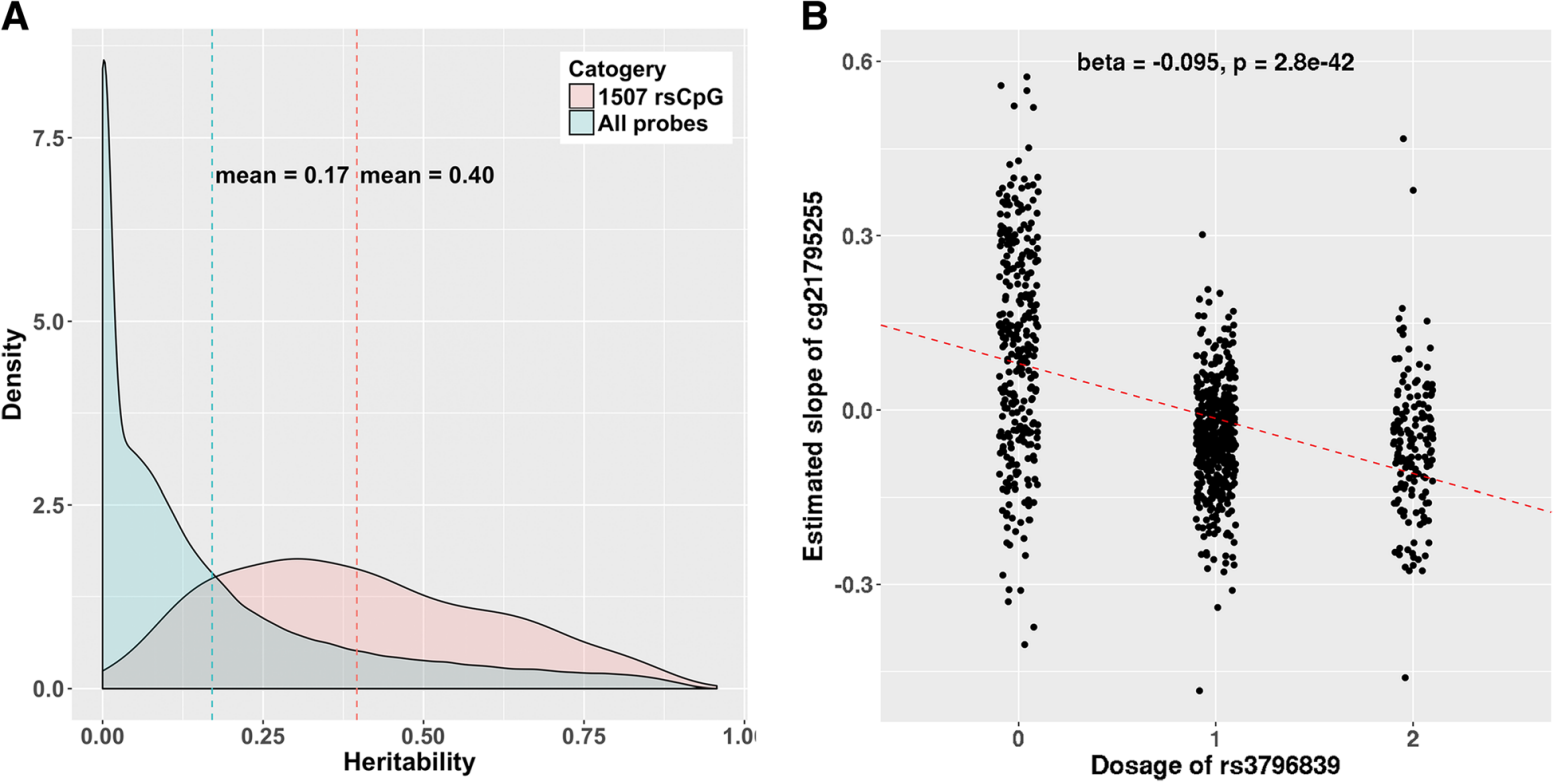
**a** The distribution of estimated heritability of 1507 rsCpGs and all probes. The heritability of rsCpGs is normally distributed, with a mean of 0.40 (SD = 0.21). It is significantly larger (*P* < 3.3 × 10^−5^, permutation test, 30,000 times) than the overall level. No significant correlation (*R* = − 0.005, *P* = 0.27) was found between heritability of probes and the distance to their meQTLs [47]. However, there is a small but significant association (*R* = 0.07, *P* < 2.2 × 10^−16^) between the heritability and the mean phenotypic correlation (*R*^2^) between a target probe and other probes on the same chromosome. This indicated that CpG sites with substantial heritability could contribute to the estimation of heritability of other CpG sites that they correlate with. **b** An example to show the significant association between SNP dosage and the random slope of DNA methylation

**Fig. 4:**
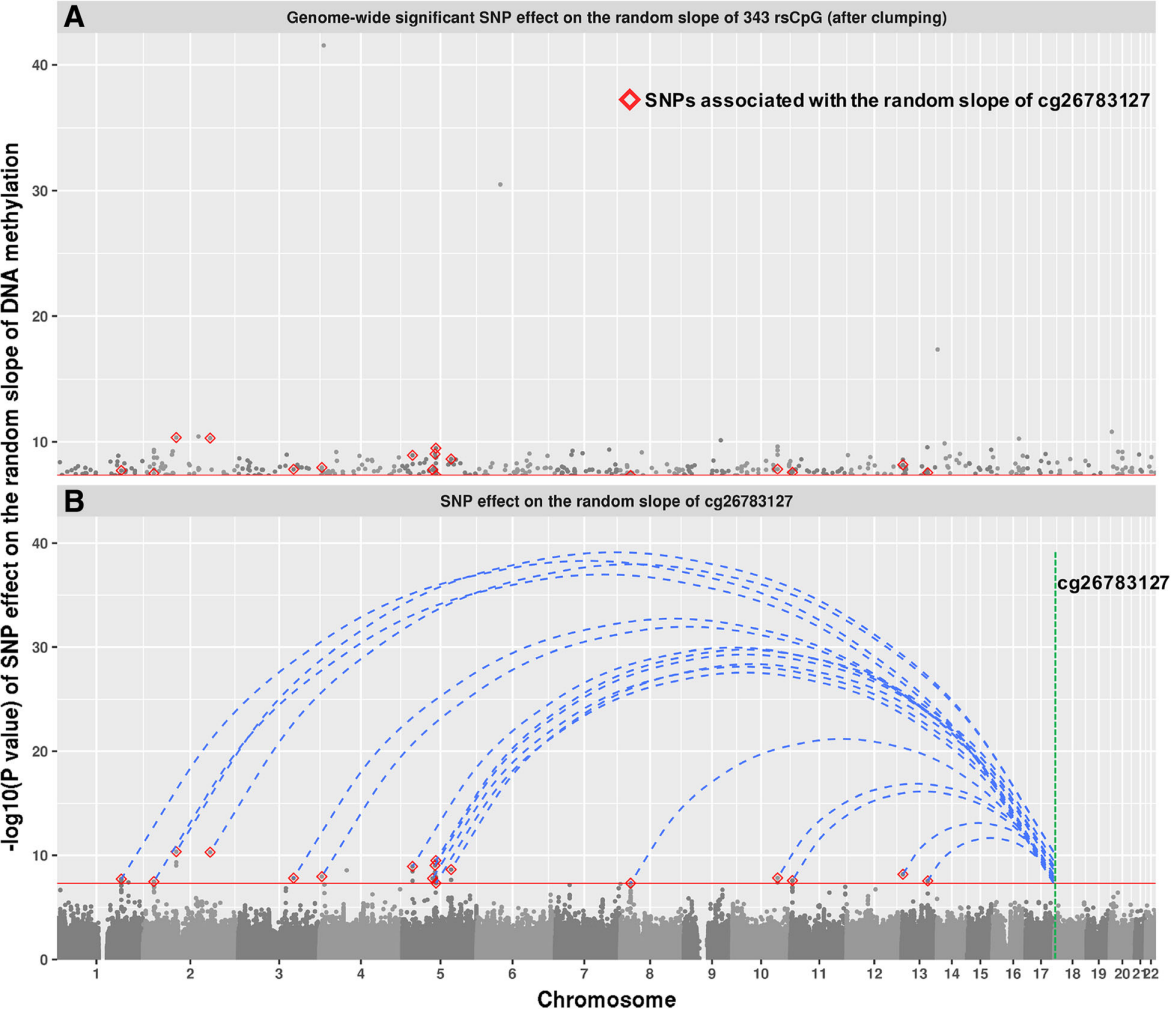
**a** The distribution of SNPs with a significant (*P* < 5 × 10^−8^) effect on the random slope of DNA methylation. The 14 SNPs associated with the random slope of cg08773226 (with the largest number of associated SNPs) are marked as diamonds. **b** The Manhattan plot to show the GWAS result on the random slope of cg08773226

rsCpGs were found to be enriched in the CpG sites with large heritability (*p* < 3.3 × 10^−5^, permutation test, 30,000 times, ‘Methods’, Fig. 3a), indicating a substantial genetic component to their variation. To examine the genetic contribution on the random effects of rsCpGs, we performed genome-wide association studies (GWASs) using PLINK2 [27], fitting the predicted random slope for each person (obtained from the mixed model analysis) as the dependent trait (‘Methods’). Results showed that there were four significant SNP-probe pairs in total (*P* < 5 × 10^−8^/1507, after linkage disequilibrium (LD) clumping, ‘Methods’), three of them are *cis* (in same chromosome) (Table 4, Fig. 3b). In addition, 343 rsCpGs were identified to have at least one genome-wide significant (*P* < 5 × 10^−8^, after LD clumping) SNP effect (436 SNP-probe pairs). Ninety-five percent of the SNPs are on different chromosomes from their corresponding probes (Fig. 4, Additional file 6: Table S5). The SNP effect on the random slope can also be interpreted as the SNP by age effect on DNA methylation (‘Methods’, Additional file 2: Figure S8). Van Dongen et al. reported 71,894 CpG sites to have an interaction between genetic effects and age (*P* < 0.05) on DNA methylation [5], and the 343 rsCpGs with a significant SNP effect on the random slope from our analyses were enriched (*P* < 3.3 × 10^−5^, permutation test, 30,000 times) in these probes (Additional file 2: Figure S9). This provides an independent confirmation of our results.

**Table 4:** Four SNPs with significant (*P* < 5 × 10^−8^/1507) effects on the random slope

SNP ID	SNP CHR	SNP POS	Probe ID	Probe CHR	Probe POS	Beta	SE	*P* value
rs3796839	4	10009917	cg21795255	4	10009916	− 0.095	0.0066	2.8 × 10^−42^
rs10948674	6	51978145	cg26820259	6	51953096	0.081	0.0067	3.2 × 10^−31^
rs190148485	20	4776083	cg24804768	12	754911	0.089	0.013	1.5 × 10^−11^
rs8015861	14	22372304	cg12819537	14	22372304	0.046	0.0053	4.6 × 10^−18^

### Relationship between rate of DNA methylation change and covariates

To detect a possible contribution of the covariates in estimating the random slope of DNA methylation, we investigated two covariates that were previously identified to have a change of variation with ageing: body mass index (BMI) and walking speed (the time to walk 6 m) [20, 40]. We fitted each of the covariates in the full model and re-estimated the significance of the variance of random slope. No significant changes were observed (Additional file 2: Figure S10), implying no contribution of these two covariates to variation of the random slope.

## Discussion

We estimated the variation in the rate of change of DNA methylation for each probe by implementing a mixed linear model in a longitudinal analysis. One thousand five hundred seven probes (rsCpGs) were found to have statistically significant variation in the rate of change between individuals. These rsCpGs were enriched in the shore region of CpG island, which is consistent with more dynamicity of DNA methylation in CpG island shore region than the CpG island itself [37].

We found that the closest genes of rsCpGs were enriched in the Homeobox gene cluster, which was also reported in Slieker et al. to be associated with age-related variably methylated positions (aVMP) in *cis* [14]. The Homeobox gene cluster is involved in the process of cell development [41], and recent evidence showed that it is related to ageing [42, 43]. Pathway analysis on these genes in PANTHER database indicated they were enriched in Wnt signalling pathway (P00057), which was also reported to be related to the ageing process [44, 45]. One of the most significantly over-represented Gene Ontology (GO) category in these genes was the developmental process (GO 0032502), which was discovered to be significant for the probes that consistently drift among twins over time [16]. These results indicate the rsCpGs may be involved in the developmental process (such as ageing) by regulating their nearby genes.

There is a significant higher heritability of the 1507 rsCpGs compared to the overall level. GWAS results on the random slope identified 436 SNP-probe pairs (343 rsCpGs) with a genome-wide significant association (*P* < 5 × 10^−8^), suggesting a SNP by age effect on the CpG sites. Among them, 95% of the SNPs were on different chromosomes from their probes, which (in the absence of non-identified confounders) indicated a potential major *trans* SNP by age effect on DNA methylation of rsCpGs.

Our study has several limitations. Although the permutation test indicates our results will not be inflated by the violations of the assumptions of the distribution of the test statistic under the null hypothesis, our results could be inflated by unknown confounding factors. We adjusted for known possible confounders, including the chronological age at which the samples were taken, but cannot exclude the possibility of unknown confounders that have effects on the mean or variance of the measured DNA methylation. The effects of covariates including age, sex and cell counts were adjusted in our quality control steps, and we further confirmed that BMI and walking speed have no effects on the rate of change in DNA methylation. However, other exposures, like medication, smoking status and disease status may potentially contribute to this variation, which can influence the estimation of random effects. Nevertheless, the 1507 rsCpGs that have a statistically significant random slope in two separate groups of individuals indicate that these results should be robust. There was no gene expression data on the same individuals available, and we simply assume that the expression of a gene can be regulated by its closest DNA methylation CpG site, which may not be true. Finally, our results are based on older individuals and may not apply to different age ranges.

## Conclusions

Ageing is strongly correlated with changes in DNA methylation, and the rates of change over time at one CpG site can differ between individuals. We detected CpG sites with different changing rates (random slope) using a mixed linear model and found 1507 CpG sites that have a statistically significant rate of change in methylation between individuals, and that these different rates of change can be partially explained by genetic effect. Genes around rsCpGs were enriched in Homeobox gene clusters and Wnt signalling pathway, both of which have been reported to be involved in the ageing process. Our results imply that the changing rate of DNA methylation varies between individuals at several CpG sites, and this difference is associated with genetic factors. These CpG sites might be useful markers to better understand individual differences in ageing.

## Additional files

Additional file 1: Quality control steps for DNA methylation. **Table S1.** Summary information for the age of all samples. **Figure S2.** Comparison of mean DNA methylation for duplicates between sets. **Figure S3.** Comparison of mean adjusted DNA methylation for duplicates between sets. (DOCX 614 kb)

Additional file 2: **Figure S1.** The distribution of match rates of control probes between and within individuals. **Figure S4.** Q-Q plot for *P* values from the detection of the random slope. **Figure S5.** The distribution of *λ*_mean_ from 500 permutation tests. **Figure S6.** Quantifying the effect of *t*^2^ and covariance between the random slope and random intercept on the estimation of random effects. **Figure S7.** The comparison of standard deviation (SD) of DNA methylation between 1507 rsCpGs and 1507 randomly selected CpG sites in each wave. **Figure S8.** Power comparison between two methods in detecting associations between SNP and DNA methylation change. **Figure S9.** The distribution of *P* value of the interaction effect between age and genetic effects from Van Dongen et al. [5] of two probe sets. **Figure S10.** The comparison of the significance of the variation of DNA methylation rate of change before and after fitting BMI and walking in the model. (DOCX 2137 kb)

Additional file 3: **Table S2.** Probes with a significant random slope in two groups of different individuals. Probe id, chi-square and *P* value were provided. (XLS 258 kb)

Additional file 4: **Table S3.** Gene Ontology analysis on the genes around the 1507 rsCpGs. (CSV 3 kb)

Additional file 5: **Table S4.** Pathway analysis on the genes around the 1507 rsCpGs in ConsensusPathDB database. (CSV 16 kb)

Additional file 6: **Table S5.** Four hundred thirty-six SNPs with genome-wide significant (*P* < 5 × 10^−8^) effects on the random slope of 343 rsCpGs. (CSV 27 kb)

## Abbreviations

BMI: Body mass index
BSGS: Brisbane Systems Genetics Study
DMR: Differently methylated region
FDR: False discovery rate
GEO: Gene Expression Omnibus
GO: Gene Ontology
GWAS: Genome-wide association study
LBC: Lothian Birth Cohort
LD: Linkage disequilibrium
LRT: Likelihood ratio test
SNP: Single nucleotide polymorphism
