# Supplementary material for: Genotype effects contribute to variation in longitudinal methylome patterns in older people
Source: Genome Med. 2018 Oct 22;10:75. doi: 10.1186/s13073-018-0585-7 (PMC6198530; doi:10.1186/s13073-018-0585-7)
Supplement: Supplementary file 1 — Quality control steps for DNA methylation. Table S1. Summary information for the age of all samples. Figure S2. Comparison of mean DNA methylation for duplicates between sets. Figure S3. Comparison of mean adjusted DNA methylation for duplicates between sets. (DOCX 614 kb) [file 13073_2018_585_MOESM1_ESM.docx]

**Raw Data**

The data set we used are from the Lothian Birth Cohort 1921 (LBC1921) and Lothian Birth Cohort 1936 (LBC1936). DNA methylation of these samples was measured using Illumina Infinium HumanMethylation450K BeadChip arrays at three time points, named set one, set two and set three, respectively. There are 2195, 996 and 552 samples in each set, and each sample has 485,512 probes measured before applying QC. In the experimental design, we used a number of duplicates between and within sets during the measurement of DNA methylation. These duplicate samples are used to quantify batch effects.

**Quality Control**

1. Duplicates removal in set two: There are 23 duplicates (samples from the same individual and same wave) in set2, and one sample in each pair was removed according the step describes in Method part. 973 samples and 485,512 probes remained.
2. Duplicates removal between set one and set two: There are 123 duplicates between the two sets. We removed one of these copies by using Method 1. 108 samples were removed from set one, and 15 samples were withdrawn from set two. A total of 3045 samples and 485,512 probes remained. This set was named set12.
3. Duplicates removal in set three: There are ten duplicates in set three. We removed one sample in each pair by using Method 1. 542 samples and 485,512 probes remained.
4. There are 31 duplicates between set three and set12, and we removed one of these copies by using the step in Method part. 26 samples were removed from set three, and five samples were excluded from set12. 3556 samples and 485,512 probes remained. This set is called set123.
5. Based on set123, we removed samples and probes with low call rates (fractions of probes with a p-value smaller than 0.01), and remove XY probes (defined in IlluminaHumanMethylation450k.db). Thresholds for the call rates for samples and probes were both 95%. After this step, 3525 samples and 470,278 probes remained.
6. Probes encompassing SNPs annotated by Illumina (GEO ID: GPL13534) and probes identified as potentially cross-hybridizing[1] were removed. After this step, 3525 samples and 344,000 probes remained.
7. 54 samples were eliminated due to not having cell count information (cell count is NA). Finally, after all these QC steps, we retained 3471 samples and 344,000 probes (**Table S1**).
8. Only individuals with DNA methylation measured at two or more different time points were considered, and samples with inconsistent measurements (match rate < 0.8) of control probes within individuals were removed, leaving 2894 samples from 954 individuals. The match rate between samples was calculated based on 65 control probes on DNA methylation 450K chip.

**Method**

1. For duplicates, the fraction of p-value smaller than 0.01 was calculated for each of the two samples, and the sample with the lower fraction was removed.

**Comparison of duplicates**

There are 31 duplicates in all three sets. We estimated the mean beta value in each sample and selected top 5% of probes with the largest DNA methylation variance. We observed that difference between samples in set one is larger than that in set two and set three. One reason is that 31 duplicates in set one are measured in 27 arrays, whereas the number is six and three in set two and set three, respectively (**Figure S2**). After adjusting for the array ID and position in each array, we obtained a smaller difference between the sets (**Figure S3**).

**Reference**

1. Price, E.M., et al., *Additional annotation enhances potential for biologically-relevant analysis of the Illumina Infinium HumanMethylation450 BeadChip array.* Epigenetics & chromatin, 2013. **6**(1): p. 4.

2. Aulchenko, Y.S., et al., *GenABEL: an R library for genome-wide association analysis.* Bioinformatics, 2007. **23**(10): p. 1294-1296.

**Figures and Tables**


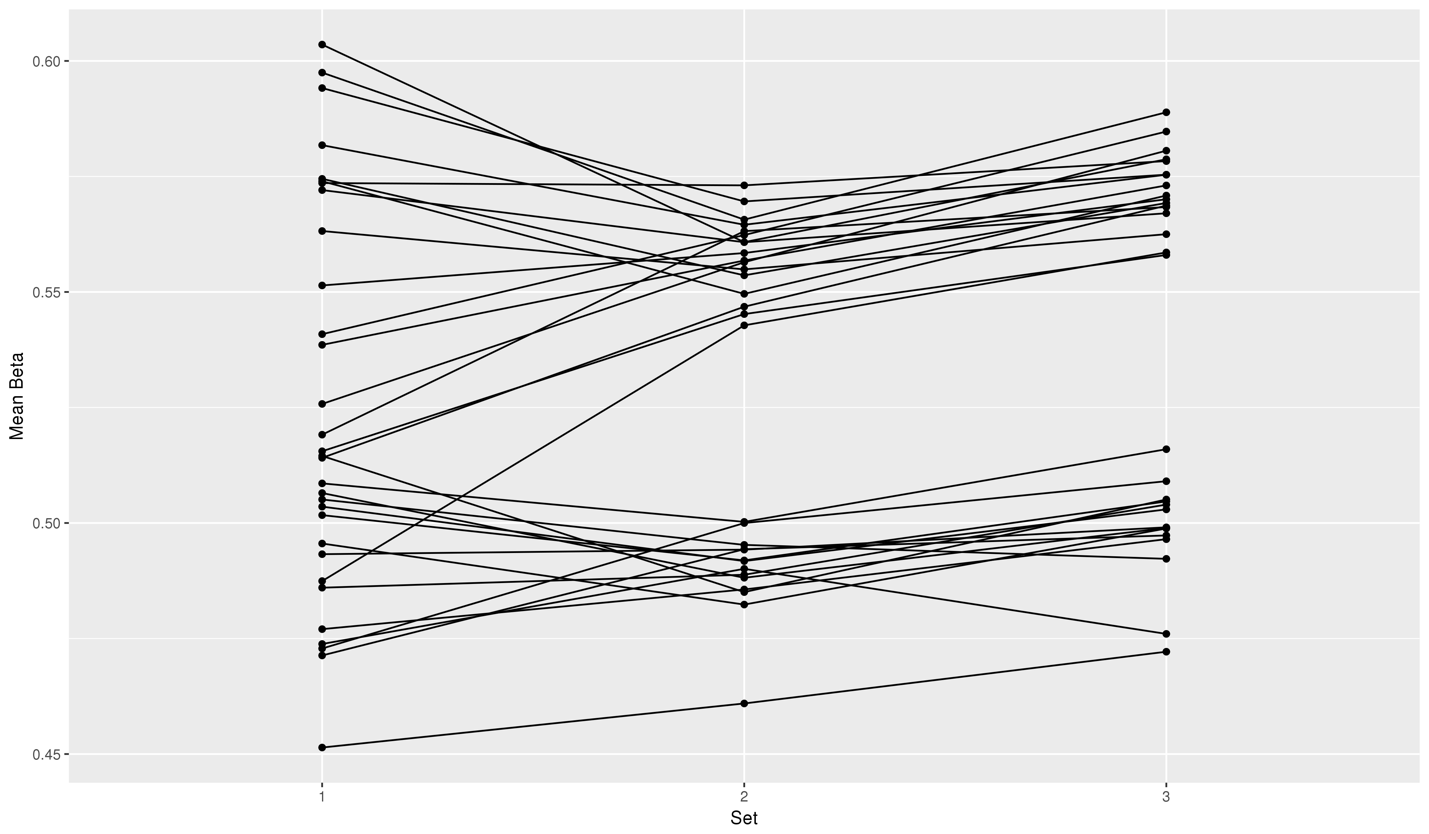


**Figure S2:** Difference of mean beta of DNA methylation for the duplicates between each set.


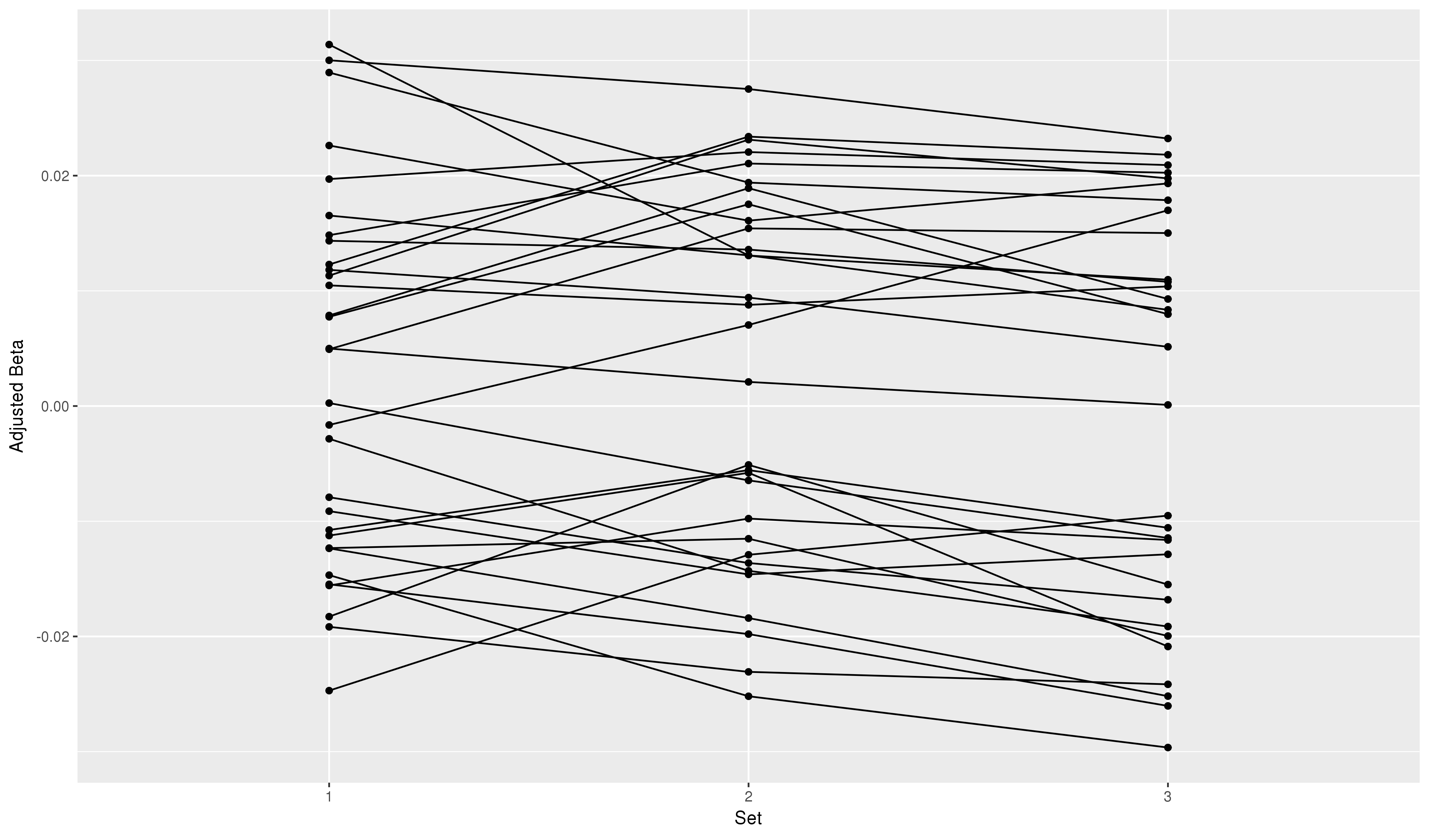


**Figure S3:** Difference of adjusted mean beta of DNA methylation for the duplicates between each set.

**Table S1:** Summary information for samples in each wave (3471 samples).

| **Cohort Wave** | **Mean Age(SD)** | **Age range** | **Female** | **Male** | **Total** |
| --- | --- | --- | --- | --- | --- |
| LBC1921W1 | 79.1(0.58) | (77.8,80.6) | 263 | 173 | 436 |
| LBC1921W3 | 86.6(0.40) | (85.8,87.5) | 93 | 78 | 171 |
| LBC1921W4 | 90.2(0.10) | (90.0,90.6) | 42 | 36 | 78 |
| LBC1936W1 | 69.6(0.83) | (67.7,71.3) | 445 | 455 | 900 |
| LBC1936W2 | 72.5(0.71) | (70.9,74.2) | 374 | 414 | 788 |
| LBC1936W3 | 76.3(0.67) | (74.7,77.7) | 288 | 315 | 603 |
| LBC1936W4 | 79.3(0.62) | (78.0,80.9) | 243 | 251 | 495 |
