# Supplementary material for: Genotype effects contribute to variation in longitudinal methylome patterns in older people
Source: Genome Med. 2018 Oct 22;10:75. doi: 10.1186/s13073-018-0585-7 (PMC6198530; doi:10.1186/s13073-018-0585-7)
Supplement: Supplementary file 2 — Figure S1. The distribution of match rates of control probes between and within individuals. Figure S4. Q-Q plot for P values from the detection of the random slope. Figure S5. The distribution of λmean from 500 permutation tests. Figure S6. Quantifying the effect of t2 and covariance between the random slope and random intercept on the estimation of random effects. Figure S7. The comparison of standard deviation (SD) of DNA methylation between 1507 rsCpGs and 1507 randomly selected CpG sites in each wave. Figure S8. Power comparison between two methods in detecting associations between SNP and DNA methylation change. Figure S9. The distribution of P value of the interaction effect between age and genetic effects from Van Dongen et al. [5] of two probe sets. Figure S10. The comparison of the significance of the variation of DNA methylation rate of change before and after fitting BMI and walking in the model. (DOCX 2137 kb) [file 13073_2018_585_MOESM2_ESM.docx]

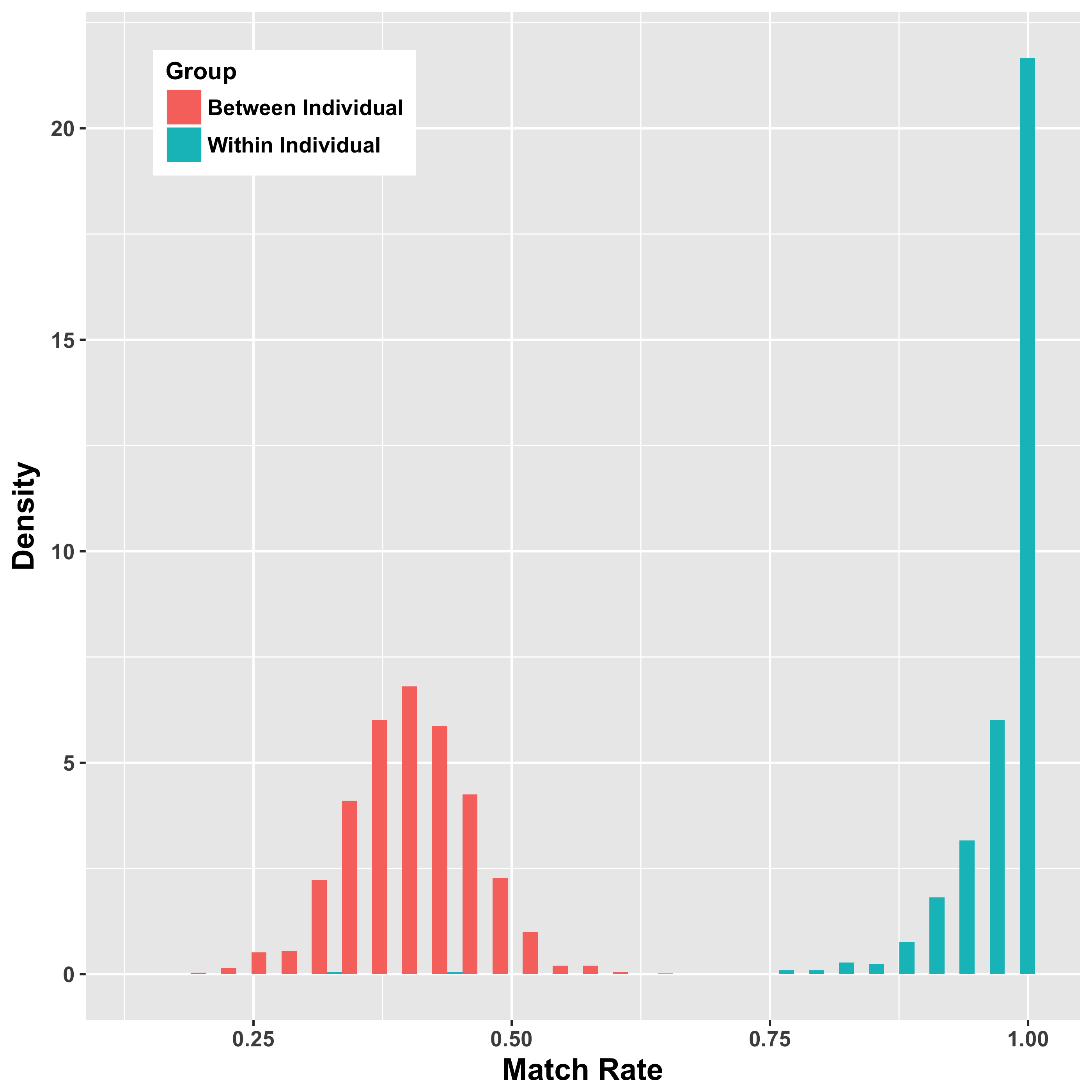


**Figure S1:** The distribution of match rates of control probes between and within individuals. Match rates were calculated based on 65 control probes on DNA methylation 450K chip.


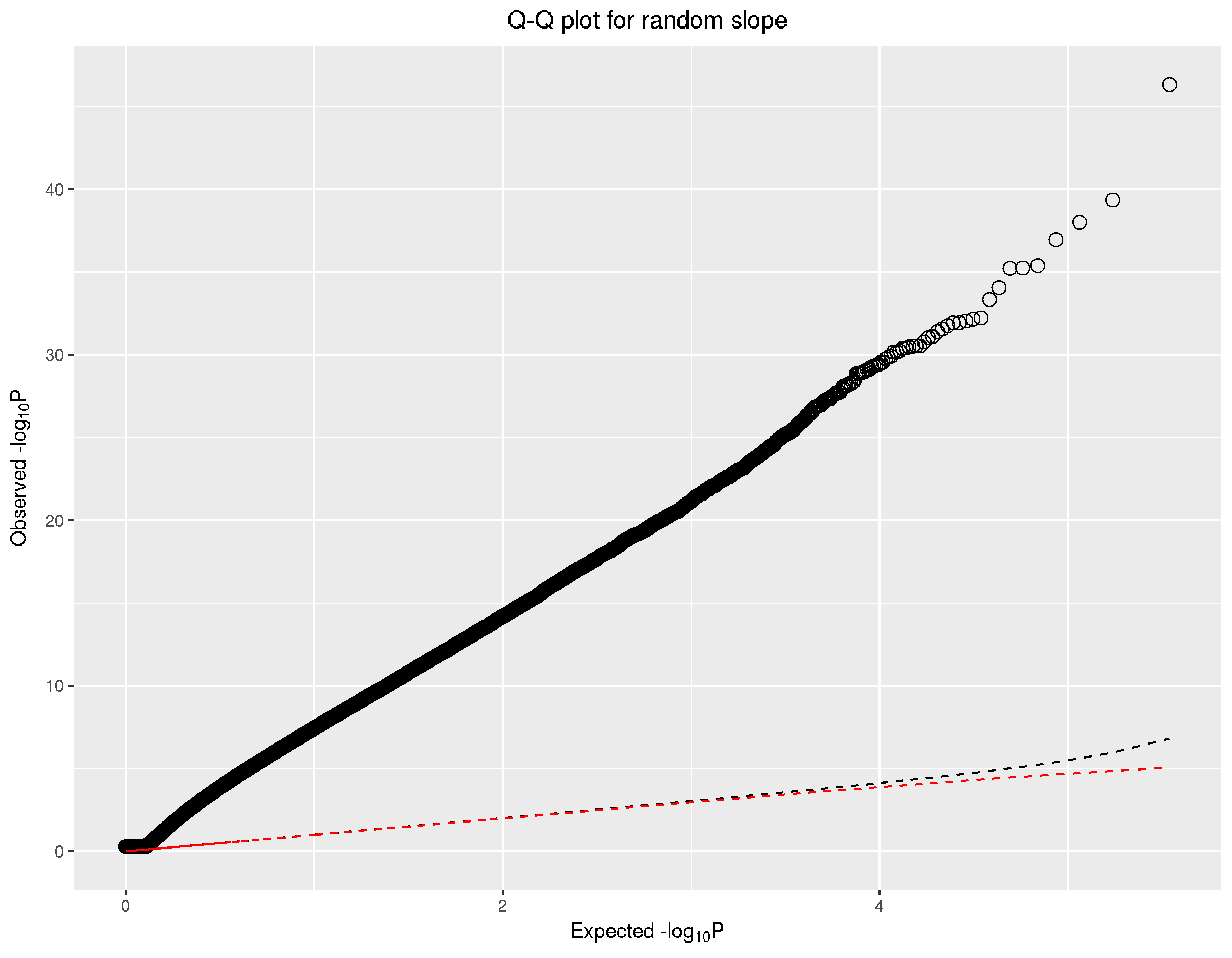


**Figure S4:** Q-Q plot for p-values from the detection of the random slope. Dash lines represent the 95% confidence interval.


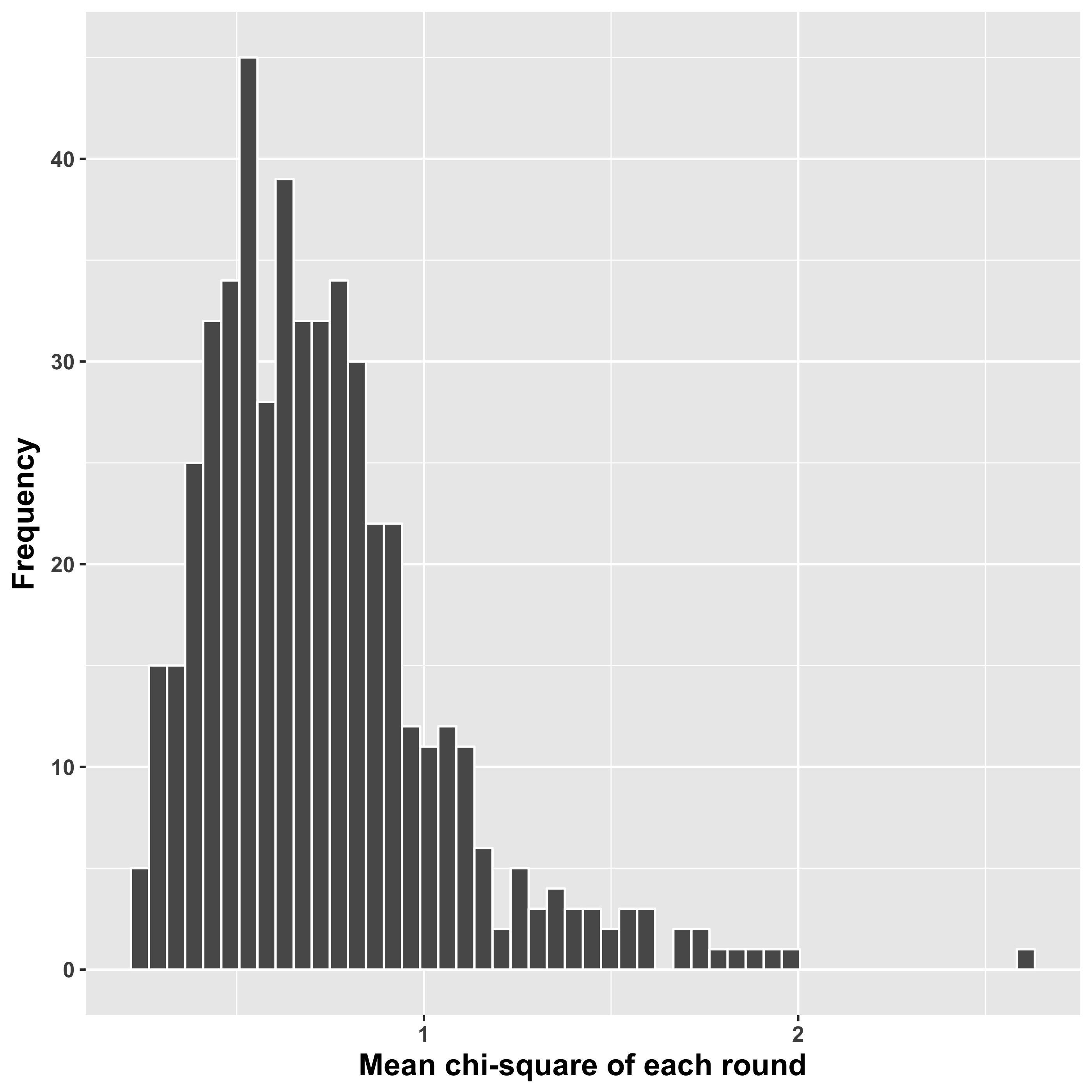


**Figure S5:** The distribution of λ_mean_ from 500 permutation tests. The mean of this distribution was around 0.73 (SD = 0.32), which shows no significant difference (P = 0.48) with the expected value of 0.5 under the null hypothesis. This indicates the statistical significance of the estimated effects of a random slope is not caused by the violations of the assumptions of the distribution of the test statistic under the null hypothesis.


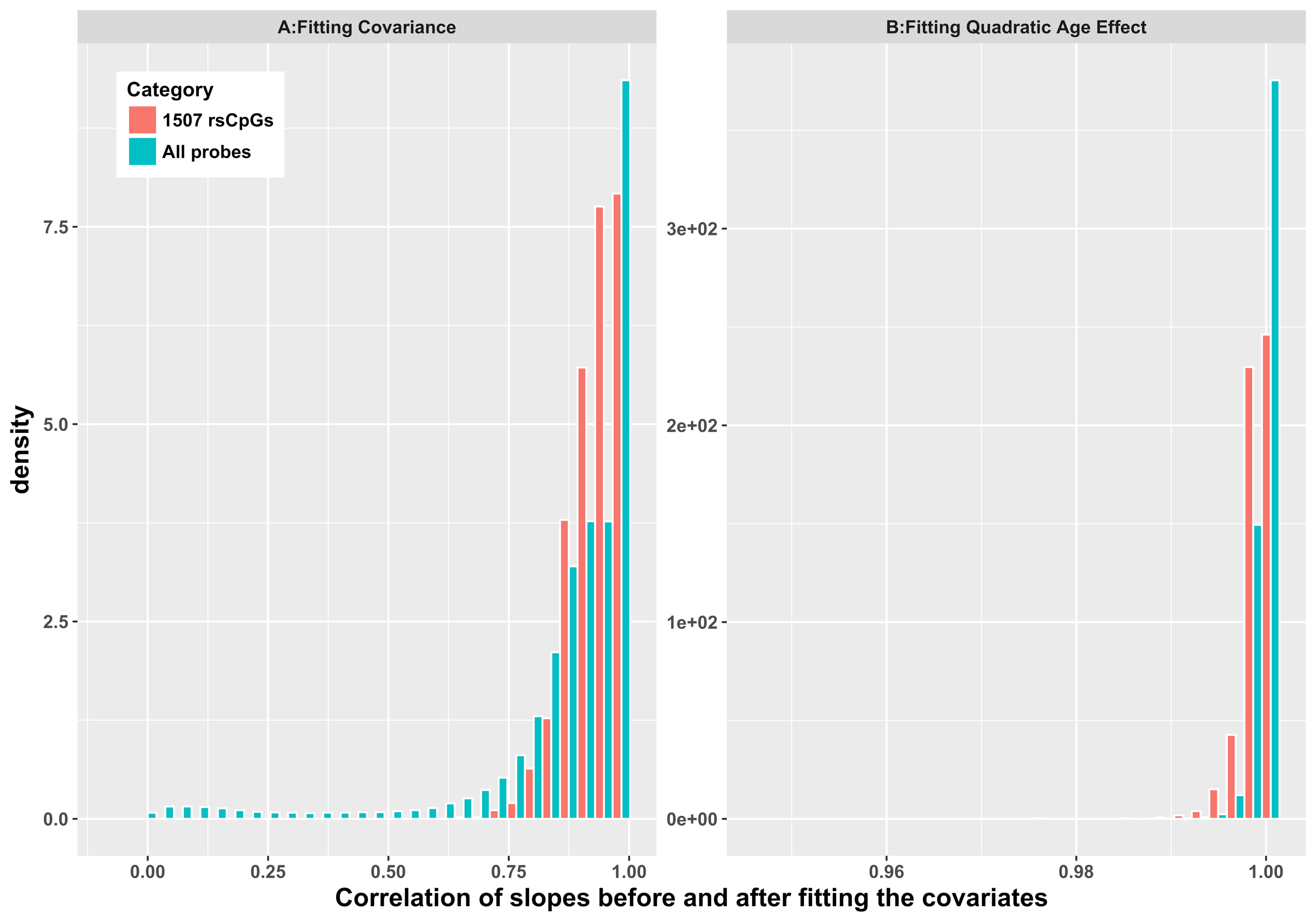
**Figure S6:** Quantifying the effect of t^2^ and covariance between the random slope and random intercept on the estimation of random effects. A: The correlation between the random slopes before and after fitting a covariance between random slope and intercept. B: The correlation between random slopes before and after including t^2^.


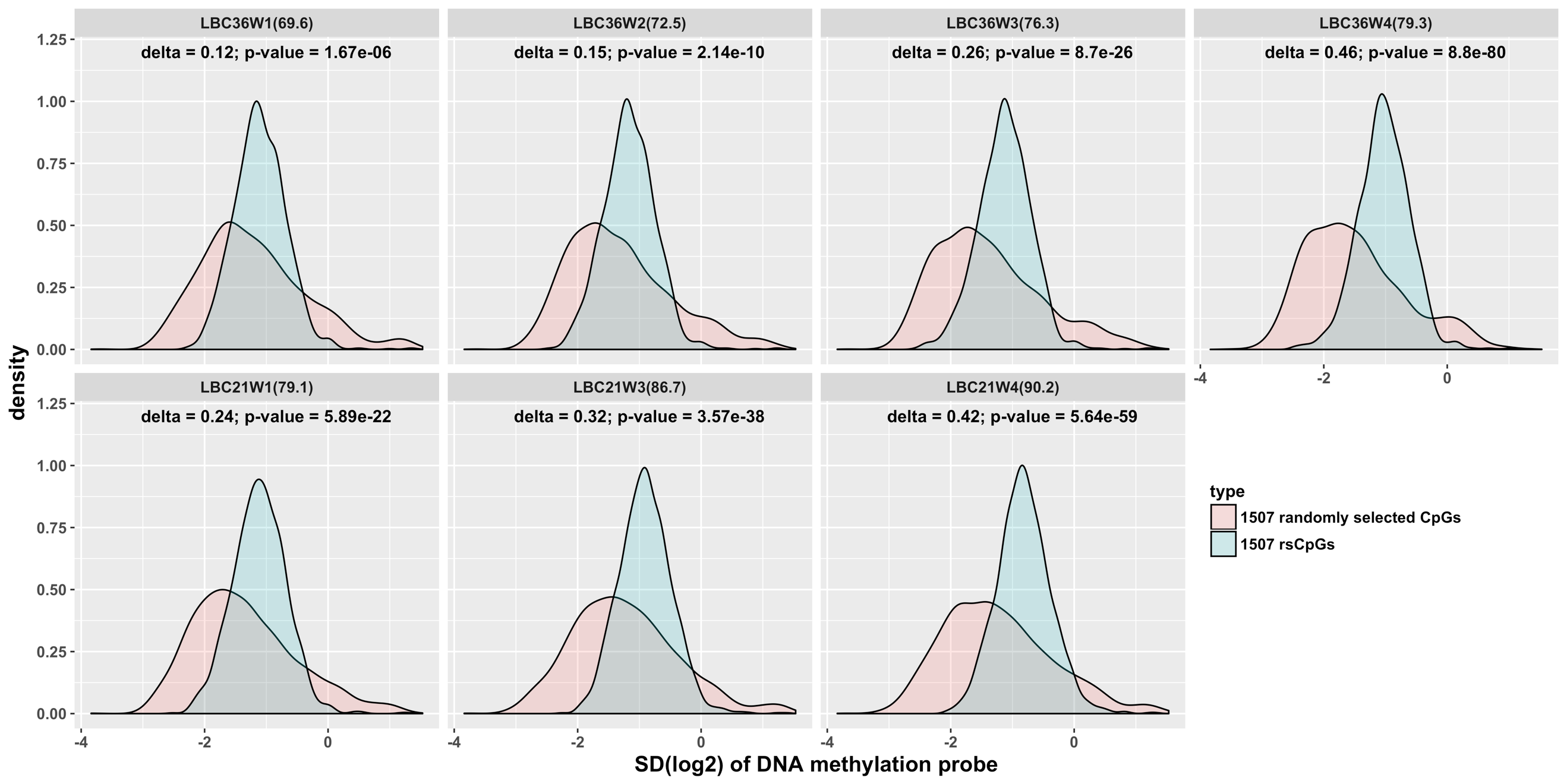


**Figure S7:** The comparison of Standard Deviation (SD) of DNA methylation between 1,507 rsCpGs and 1,507 randomly selected CpG sites in each wave. Numbers in each bracket is the mean age in this wave. Delta is the mean difference between SD (log2) from 1,507 rsCpGs


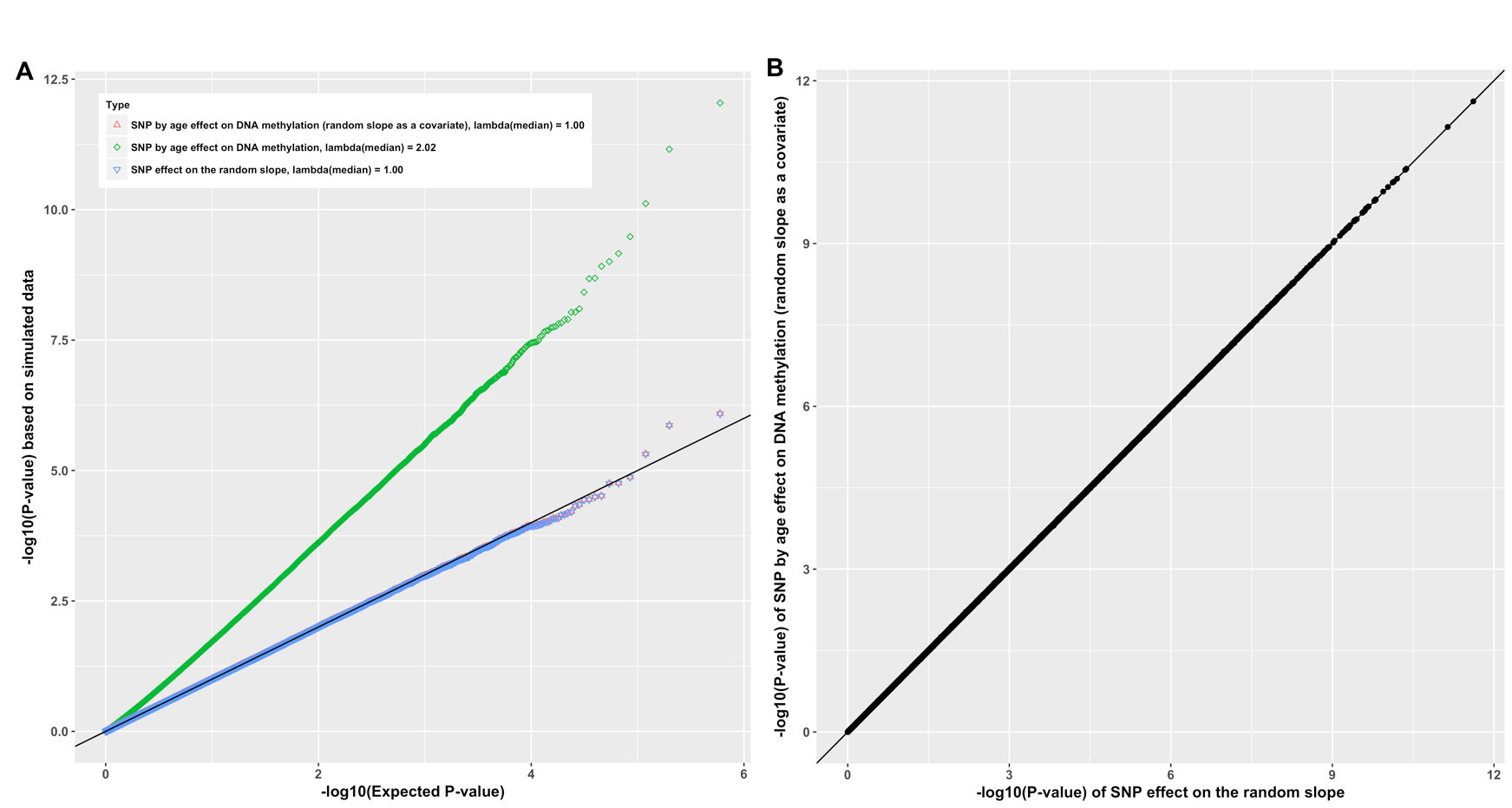


**Figure S8:** Power comparison between two methods in detecting associations between SNP and DNA methylation change. A: Q-Q plot for p-values when no SNP effect on the random slope was simulated. B: P-values in the x-axis are from the association between SNPs and random slopes, and p-values in the y-axis are from the association between SNP by age effect and DNA methylation (with random slope as a covariate). The random slope in the simulated data was associated with SNPs.


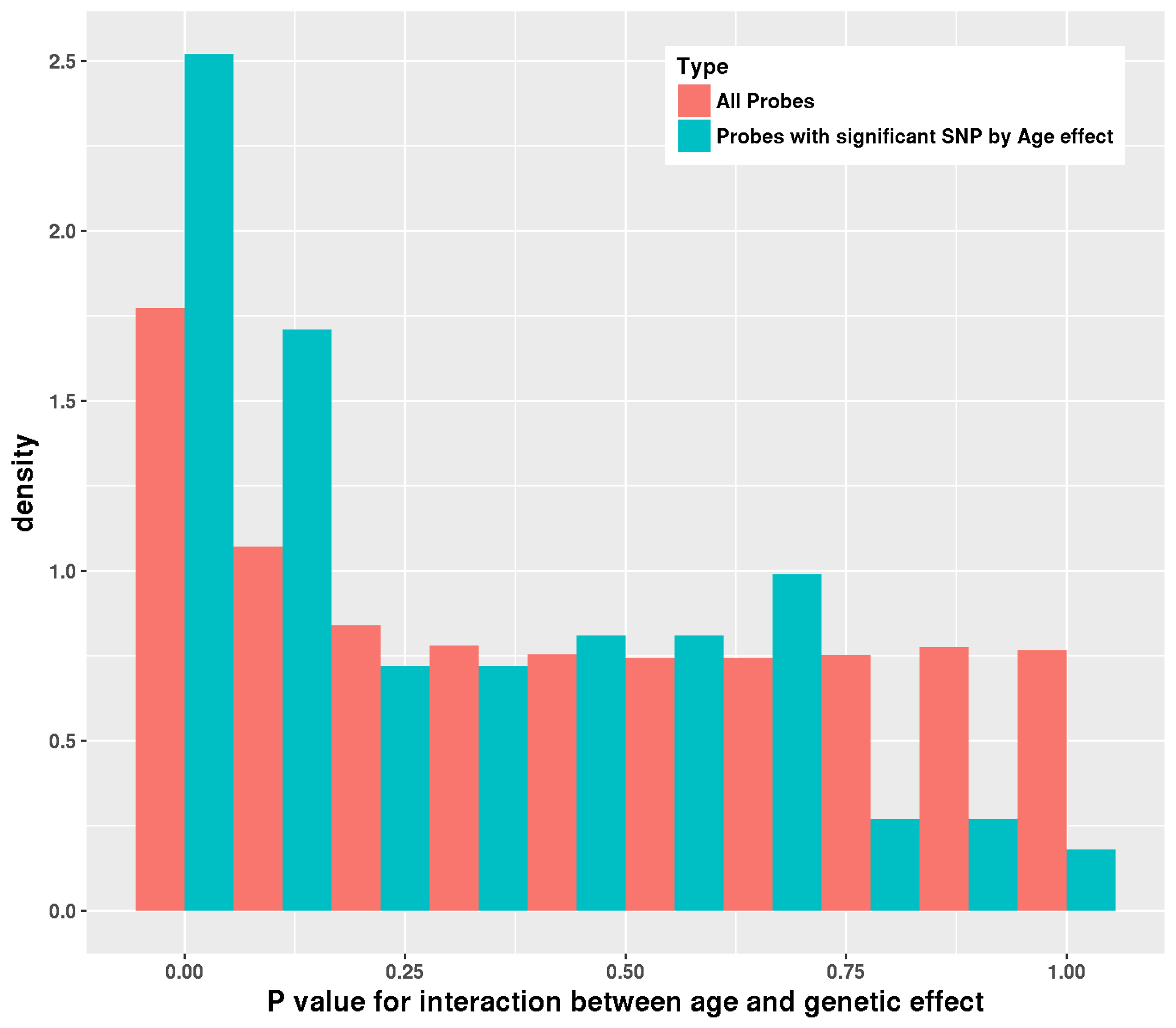


**Figure S9:** The distribution of p-value of the interaction effect between age and genetic effects from Van Dongen et al.[1] of two probe sets. One has 343 rsCpGs with a significant SNP effect on random slope in our results, the other contains all probes.


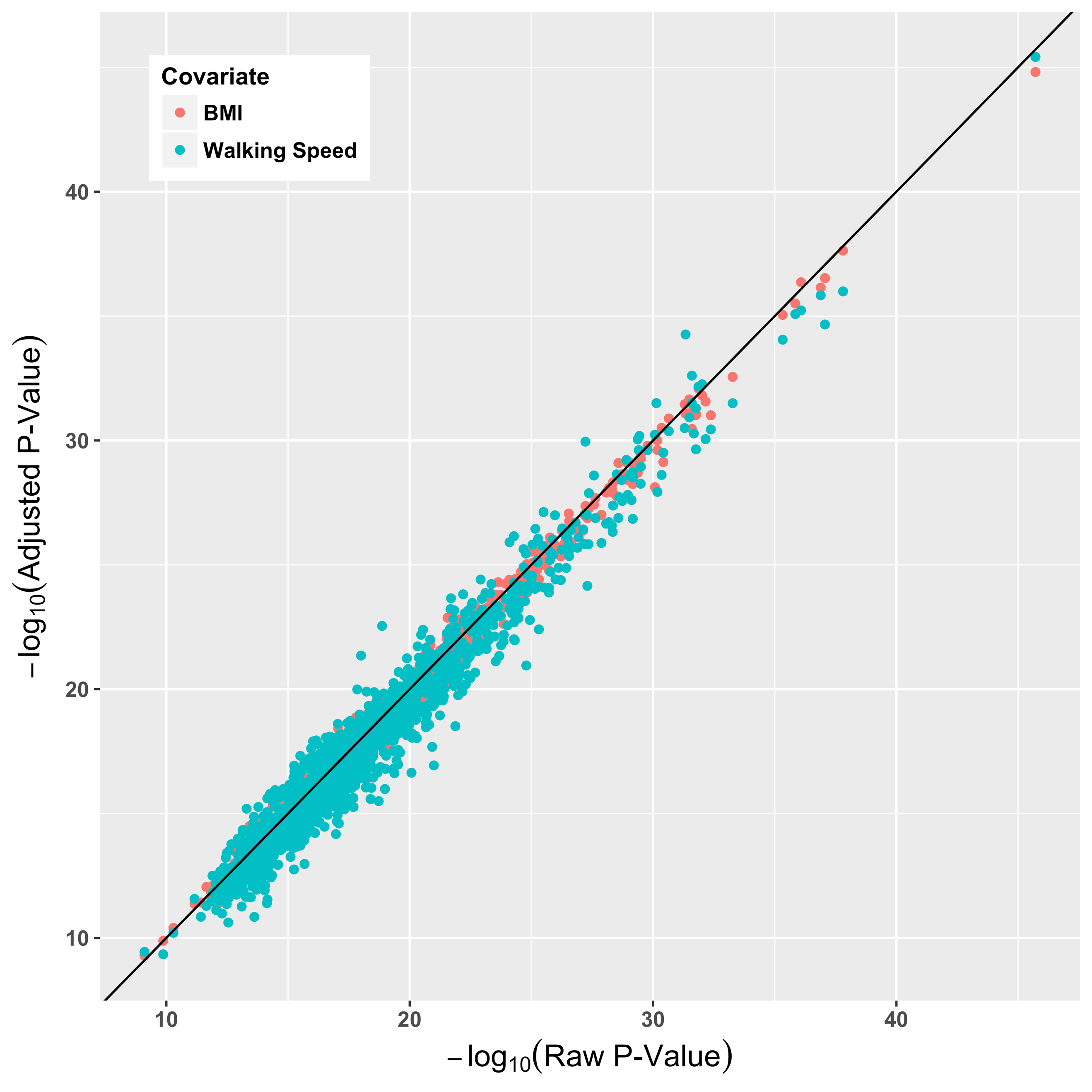


**Figure S10:** The comparison of the significance of the variation of DNA methylation rate of change before and after fitting BMI and walking in the model.

**Reference**:

1. Van Dongen J, Nivard MG, Willemsen G, Hottenga J-J, Helmer Q, Dolan CV, Ehli EA, Davies GE, Van Iterson M, Breeze CE: **Genetic and environmental influences interact with age and sex in shaping the human methylome.** *Nature communications* 2016, **7**.
